# Nutritional status and HIV in rural South African children

**DOI:** 10.1186/1471-2431-11-23

**Published:** 2011-03-25

**Authors:** Elizabeth W Kimani-Murage, Shane A Norris, John M Pettifor, Stephen M Tollman, Kerstin Klipstein-Grobusch, Xavier F Gómez-Olivé, David B Dunger, Kathleen Kahn

**Affiliations:** 1 MRC/Wits Rural Public Health and Health Transitions Research Unit (Agincourt), School of Public Health, Faculty of Health Sciences, University of the Witwatersrand, South Africa; 2 African Population and Health Research Centre (APHRC), Nairobi Kenya; 3 MRC Mineral Metabolism Research Unit, Department of Paediatrics, Faculty of Health Sciences, University of the Witwatersrand, South Africa; 4 Umeå Centre for Global Health Research, Department of Public Health and Clinical Medicine, Umeå University, Umeå, Sweden; 5 INDEPTH Network, Accra, Ghana; 6 School of Public Health, Faculty of Health Sciences, University of the Witwatersrand, South Africa; 7 Department of Paediatrics, University of Cambridge, UK

## Abstract

**Background:**

Achieving the Millennium Development Goals that aim to reduce malnutrition and child mortality depends in part on the ability of governments/policymakers to address nutritional status of children in general and those infected or affected by HIV/AIDS in particular. This study describes HIV prevalence in children, patterns of malnutrition by HIV status and determinants of nutritional status.

**Methods:**

The study involved 671 children aged 12-59 months living in the Agincourt sub-district, rural South Africa in 2007. Anthropometric measurements were taken and HIV testing with disclosure was done using two rapid tests. Z-scores were generated using WHO 2006 standards as indicators of nutritional status. Linear and logistic regression analyses were conducted to establish the determinants of child nutritonal status.

**Results:**

Prevalence of malnutrition, particularly stunting (18%), was high in the overall sample of children. HIV prevalence in this age group was 4.4% (95% CI: 2.79 to 5.97). HIV positive children had significantly poorer nutritional outcomes than their HIV negative counterparts. Besides HIV status, other significant determinants of nutritional outcomes included age of the child, birth weight, maternal age, age of household head, and area of residence.

**Conclusions:**

This study documents poor nutritional status among children aged 12-59 months in rural South Africa. HIV is an independent modifiable risk factor for poor nutritional outcomes and makes a significant contribution to nutritional outcomes at the individual level. Early paediatric HIV testing of exposed or at risk children, followed by appropriate health care for infected children, may improve their nutritional status and survival.

## Background

Achievement of two of the Millennium Development Goals (MDGs) aimed at reducing malnutrition and child mortality by 2015 will depend in part on the ability of governments/policymakers to address the health and nutritional status of all children in general and of children infected or affected by HIV/AIDS in particular. Though some gains have been made in reducing child malnutrition, millions of children are still malnourished: some 26% of children under five years suffered from malnutrition in developing countries in 2006 [1]. Malnutrition is a risk factor for poor cognitive development, reduced human capital, premature death and other health consequences [2-4]. HIV/AIDS, which is highly prevalent in sub-Saharan Africa [5], may complicate child malnutrition in settings with high HIV prevalence such as South Africa.

HIV/AIDS is associated with nutritional deficiencies in infected children [6] while undernutrition influences disease progression, increases morbidity and lowers survival of HIV infected persons [7]. Additionally, HIV/AIDS has enormous impact on food security of affected households [8,9]. Other covariates of child malnutrition have been documented including child level factors such as age and birth weight; maternal level factors such as maternal age and education; household level factors such as food insecurity and social economic status; and community level factors such as sanitation and environmental factors [10-12]. Importance of these factors to nutritional status of children may vary with differing contexts, indicating the need for context-specific evidence.

South Africa, like neighbouring Southern African countries, is experiencing one of the most severe HIV epidemics in the world [5]. Nationally, about a third of pregnant women visiting public antenatal clinics are HIV infected [13]. Additionally, there are high levels of food insecurity at household level: about 35% of the total population lack food security, a vulnerability aggravated by HIV/AIDS in South Africa [14]. Malnutrition remains highly prevalent in South Africa particularly in rural areas [15]. It is against the backdrop of the dual burden of high HIV prevalence and high risk of malnutrition in South Africa, that this study was conducted.

The study investigates the prevalence of HIV infection among 12-59 months old children, patterns of nutritional status by HIV status, and determinants of malnutrition in this age group. Technicalities of HIV testing of young children and ethical issues limit evidence on nutritional status of HIV positive children in randomly selected samples at population level; this is a key strength of this study. Our study thus makes an important contribution in establishing the wellbeing of HIV positive children in a community setting.

## Methods

### Study Setting and Population

This study was conducted in the rural Agincourt sub-district of Mpumalanga Province, northeast South Africa, which borders Mozambique. It was nested within the Agincourt health and socio-demographic surveillance system (Agincourt HDSS), established in 1992 which covers the entire sub-district and follows some 70,000 people living in 11,700 households in 21 contiguous villages. The population comprises Tsonga-speaking people, some 30% of whom are of recent Mozambican origin, having entered South Africa mainly as refugees in the early to mid-1980s during the civil war in Mozambique. The area is dry with household plots too small to support subsistence farming. The area is characterised by high levels of poverty; the province in which the study area is located has one of the highest poverty rates in South Africa, at 64% [16]. Labour migration is widespread involving up to 60% of working-age men and growing numbers of women [17,18]. A network of five primary care clinics refers to a larger public health centre; the nearest district hospital is 25 kilometers away. Over a third of pregnant women visiting public health clinics in the area are HIV positive [13].

### The Agincourt Health and Demographic Surveillance System (HDSS)

The Agincourt HDSS is a multiround prospective community study and involves systematic annual recording of all births, deaths and migration events occuring in Agincourt since 1992. Individual characteristics including date of birth, sex, and nationality of origin are recorded. Additional data are collected as special census modules nested within the annual update rounds. These include education, child social grant uptake, union status and food security. An asset survey conducted in each household every two years gives a measure of household socioeconomic status. Detailed information on the Agincourt HDSS is provided elsewhere [19,20].

Explanatory variables used in this study were obtained from the Agincourt HDSS: child's age and sex; birth weight; place of delivery; child's relationship to household head; age, nationality, highest education level, and marital/union status of the mother; mother's co-residence with child; age, sex and highest education level of household head; household food security and socio-economic status; and village of residence.

The food security data utilised in this study was collected in 2007 as a panel survey nested within the Agincourt HDSS. Food insecurity was defined as not having reported enough food to eat either in the last one month or in the last one year, whereas food secure households were characterized as households reporting sufficient to eat both in the last one month and one year respectively.

Household wealth index was constructed from the 2007 survey [21], which documented type and size of dwelling; water and sanitation facilities; electricity; modern assets such as fridge and television; transport assets such as bicycle and car; communication assets such as cellphone; and livestock such as cattle. A summated absolute score for the wealth indicator was constructed. To begin, each asset variable was coded with the same valence (i.e. increasing values correspond to greater socio-economic status (SES)) and effectively given equal weight by rescaling so that all values of a given asset variable fall within the range (0, 1). Assets were then categorized into five broad groups - 'modern assets', 'livestock assets', 'power supply', 'water and sanitation' and 'dwelling structure'. For each household within each asset group, the rescaled asset values were summed and then rescaled again to yield a group-specific value in the range (0, 1). Finally for each household, these five group-specific scaled values were summed to yield an overall asset score whose value could theoretically fall in the range (0, 5). Household wealth tertiles were generated from the absolute SES score using the Stata's xtile command and labelled as most poor (lowest 1/3), middle class, and least poor (highest 1/3).

### Growth Survey

We conducted a cross-sectional anthropometric survey between April and July 2007. This survey targetted 4000 children and adolescents aged between 1 and 20 years, who were permanent residents in the study area at the time of sampling and had lived in the study area at least 80% of their lives, since birth or since 1992 when the Agincourt HDSS started. The sample size was determined so as to get sufficient numbers per age and sex group. A total of 2000 girls and 2000 boys were targetted, 100 participants in each age and sex group. Over-sampling by 10-15 children per age and sex group was done to cater for possible non-participation of the target sample. A total of 4658 children were therefore sampled. The study sample was randomly selected from the Agincourt HDSS database described above, with the child as the sampling unit as follows: all the children aged 1-20 years in the database, meeting the inclusion criteria, were allocated a unique identity number, stratification was then done by age, sex and village and a random sample using the unique identity number was drawn from each strata proportionate to the population size of the village. More details on the sample can be obtained from a previous publication [22]. The time between the most recent database and study fieldwork resulted in no participants under the age of one year. Hence for the purpose of this paper, children aged 12-59 months were included; a total of 671 children.

Invitation to participate was initiated during a home visit at which informed consent to participate in the growth survey was obtained from the caregiver (caregiver refers to mother or non-mother caregiver who was the usual carer of the child). Data were collected during weekends in schools centrally located within the study villages. Anthropometric measurements included height and weight: height was measured using a stadiometer (Holtain, UK) calibrated in millimeters. For all children aged less than 24 months, length was measured using an inelastic tape measure in a recumbent position on a flat surface. Weight in kilograms (to one decimal point) was determined using a mechanical bathroom scale (Hanson, UK). All anthropometric measurements were taken according to standard procedures [23]. To standardise measurements and enhance quality, each measurement was conducted by a dedicated lay fieldworker specifically trained in the technique by experts in the field.

Height-for-age z-scores (HAZ), weight-for-age z-scores (WAZ), and weight-for-height (WHZ) z-scores were generated using the 2006 WHO standards, through use of the WHO Anthro 2005 program, Beta Version [24]. The 2006 WHO standards were developed following the Multicentre Growth Reference Study (MGRS) commissioned by the WHO, implemented between 1997 and 2003. The study was conducted internationally in diverse countries including Brazil, India, Ghana, Norway, Oman and the USA. The study involved healthy breastfed children that were raised in conducive environments that enhance full growth potential [25,26]. The 2006 WHO standards are recommended for international use, and this is justified further by earlier studies that indicated that the growth of young children is similar across different ethnic backgrounds [27]. Stunting, underweight and wasting were determined as z-scores <-2 respectively [28].

HIV testing of children aged 12-59 months was carried out after anthropometric measurements were taken. Informed consent for the HIV test was obtained separately from the earlier consent for anthropometric measurements. Pre-test counselling was given to the caregiver of the child before the test was done. Blood was obtained from each child through a finger prick. Two rapid tests: Uni-Gold™ (Trinity Biotech, Bray, Ireland) and Determine™ (Abbott, Wiesbaden, Germany) were performed concurrently in accordance with WHO recommendations for HIV screening in children [29]. Of the 671 children aged 12-59 months in the study, 640 consented to testing, a 95% response rate. A child was defined as HIV negative if both tests were negative, HIV positive if both tests were positive, and indeterminate if either of the tests was negative and the other positive. The result was given to the caregiver if s/he wished to know the status. All but two respondents requested the result; one refused and one could not wait for the result due to other commitments but was informed later. All caregivers of HIV positive children and those with indeterminate results (only one) were counselled and referred for a confirmatory test, further counselling and other support at the nearest primary health care facility. Similarly, all children identified as malnourished were referred to local clinics for nutritional advice and support; these had been fully briefed on the study.

### Ethical Clearance

Ethical clearance for the two studies was granted seperately by the University of the Witwatersrand Committee for Research on Human Subjects, (Medical). Ethical clearance number for the 2007 growth survey is M070244, while for the Agincourt HDSS is M960720. Informed consent was obtained from the children's caregivers.

### Data Analysis

Data analysis using Stata version 10.0 (StataCorp LP, College Station, Texas, USA) describes by HIV status, mean HAZ, WAZ and WHZ (and their standard deviations), as well as prevalence of stunting, underweight and wasting. T-test and oneway ANOVA were used to test for statistically significant differences in means by HIV status, while Chi-square test was used for proportions.

Univariate linear and logistic regression were done to investigate the association of independent variables, listed above, to nutritional outcomes. Categories for covariates such as birth weight and mother's/household head's age, were created for ease of interpretation with regards to policy implications. Missing values for each variable were allocated an independent category in the regression models to maintain all participants in the analysis. The variables affected include birth weight obtained from the Road-to-Health card (22% missing values), household head education (10% missing values), mother's education (7% missing values), food security (6% missing values), socio-economic status (2.5% missing values), and mother's age, mother's nationality and household head relationship to child (<2% missing values each). Explanatory variables found to be significantly associated with the outcome variable at the 10% level of significance in the univariate analysis were included in the multivariate linear and logistic regression analysis. HIV status was maintained in all the models, being the key predictor of interest. Bias due to non-response was examined using data from Agincourt HDSS whereby comparisons between participants and non-participants were made using chi-square test and t-test. A p-value of <0.05 was considered statistically significant in all the analysis.

## Results

This study involved 671 children aged 12-59 months in 2007: 338 (50.4%) boys and 333 (49.6%) girls. About a quarter of sampled children aged less than five years did not participate in the study. Comparisons of demographic variables between participants and non-participants are shown on Table 1. For most of the demographic variables included, there were no significant differences. However non-participants were significantly younger, had younger mothers and were more likely to have a grand-parent as the head of household (p < 0.05, respectively). (Table 1)

**Table 1:** Comparison of participants' and non-participants' characteristics (n = 902), Agincourt, South Africa (2007)

Characteristic	Non-participants (n = 231)	Participants (n = 671)	P-Value
**Child Characteristics**			
Child age in years [mean (SD)] ^1^	2.3 (1.1)	2.5 (1.1)	**0.012**
Child Sex (%)			
Boys	48.9	50.2	0.732
Girls	51.1	49.8	
Birth weight [mean (SD)]	3.0	3.1	0.161
Birth weight categories (%)			
Low; <2.5 kg	9.4	10.3	0.706
Normal; 2.5 kg	90.6	89.7	
**Maternal Characteristics**			
Mother's age (%)			
15-24	41.6	31.4	
25-34	40.7	42.0	**0.005**
35+	17.7	26.2	
Mother's Nationality (%)			
South African	68.6	66.1	0.496
Mozambican	31.4	33.9	
Mother's Education (%)			
None	10.7	13.3	0.580
Less than completed secondary	65.6	62.6	
Secondary & Tertiary	23.7	24.2	
Mother Union Status (%)			
Currently in union	46.3	47.7	0.719
Not in union	53.7	52.3	
Mother co-residence			
Co-residing	96.1	96.9	0.575
Not co-residing	3.9	3.1	
Delivery place (%)			
Health facility	87.3	84.6	0.340
Home	12.7	15.4	
**Household Characteristics**			
Household head Age			
15-34	13.5	11.1	
35-49	38.0	42.4	0.406
50+ years	48.5	46.6	
Household head Sex			
Male	62.0	65.1	0.396
Female	38.0	34.9	
Household head Education			
None	47.0	41.1	
Less than completed secondary	36.4	45.8	0.061
Secondary & Tertiary	16.7	13.1	
Relationship to child			
Parent	32.9	42.7	
Grandparent	50.7	44.8	**0.026**
Other	16.5	12.5	
Food Security			
Enough	78.1	80.0	0.566
Not enough	21.9	20.0	
SES Tertiles			
Most poor	42.7	39.6	
Middle	26.4	31.7	0.336
Least poor	30.9	28.8	
**Area of Residence**			
Mainly South African village	91.3	92.6	0.554
Mainly Mozambican village	8.7	7.5	

^1^Age at the time of sampling

### Characteristics by HIV status

Of the study participants, 612 were HIV negative, 28 HIV positive, and 31 were not tested (no consent for the test), resulting in a HIV prevalence of 4.4% (95% CI: 2.79 to 5.97) in this age group; 3.1% (95% CI: 1.20 to 5.03) in boys and 5.6% (95% CI: 3.10 to 8.20) in girls. The non-participation for the test was mainly because of the decision-maker being unavailable to give consent and this was largely because they were migrant workers, working away from home.

Table 2 shows the characteristics of study participants by HIV status. The mean birth weight for all children was 3.1 kg (SD; 0.49) and 10% of children had low birth weight (<2.5 kg). HIV positive children had significantly lower mean birth weight compared to HIV negative children (p = 0.029). Mothers of HIV positive children were significantly older than mothers of HIV negative children (p = 0.029), with two-thirds being in the age category 25-34 years (67%). Mothers of HIV positive children had a significantly lower level of education compared to those of HIV negative children (p = 0.017). Twenty one mothers did not co-reside with their children (3% of the total sample, 81% of whom had died). A significantly higher proportion of mothers of HIV positive children (p = 0.018) did not co-reside with the child.

**Table 2:** Characteristics of study participants by HIV status (n = 671), Agincourt, South Africa (2007)

Characteristic	All [n = 671]	HIV- [n = 612]	HIV+ [n = 28]	Unknown status [n = 31]	P-Value [comparing all three categories]	P-value [comparing HIV- & HIV+ only]
**Child Characteristics**						
Child age in years [mean (SD)] (n = 671)^1^	3.1 [1.1]	3.1 [1.1]	3.1 [1.2]	3.3 [1.0]	0.636	0.968
Child Sex (%) (n = 670)						
Boys (n = 338)	50.3	50.8	35.7	54.8		
Girls (n = 333)	49.6	49.2	64.3	45.2	0.259	0.118
Birth weight (continuous) [mean (SD)] (n = 522)	3.1 [0.49]	3.1 [0.48]	2.9 [0.54]	3.2 [0.62]	0.062	**0.029**
Birth weight categories (%) (n = 522)						
Low; <2.5 kg (n = 54)	10.3	9.9	16.0	14.3	0.515	0.323
Normal; 2.5 kg (n = 468)	89.7	90.1	84.0	85.7		
**Maternal Characteristics**						
Mother's age (%) (n = 659)						
15-24 (n = 77)	31.4	33.0	11.1	19.4		
25-34 (n = 207)	42.0	40.1	66.7	45.2	**0.029**	**0.017**
35+ (n = 175)	26.6	26.3	22.2	35.5		
Mother's Nationality (%) (n = 658)						
South African (n = 435)	66.1	65.3	59.3	87.1	**0.033**	0.517
Mozambican (n = 223)	33.9	34.7	40.7	12.9		
Mother's Education (%) (n = 625)						
None (n = 83)	13.3	13.3	23.0	3.5		
< completed secondary (n = 381)	62.6	62.3	69.2	62.1	0.078	0.089
Secondary & Tertiary (n = 191)	24.2	24.4	7.7	34.5		
Mother Union Status (%) (n = 671)						
Currently in union (n = 320)	47.7	48.2	39.3	45.2	0.626	0.356
Not in union (n = 351)	52.3	51.2	60.7	54.8		
Mother co-residence (n = 671)						
Co-residing (n = 650)	96.9	97.2	89.3	96.8	0.062	**0.018**
Not co-residing (n = 21)	3.1	2.8	10.7	3.3		
Delivery place (%) (n = 671)						
Health facility (n = 568)	84.7	84.2	85.7	93.6	0.362	0.824
Home (n = 103)	15.4	15.9	14.3	6.5		
**Household Characteristics**						
Household head Age (n = 668)						
15-34 (n = 74)	11.1	10.8	21.4	6.5		
35-49 (n = 283)	42.4	41.7	50.0	48.4	0.191	0.076
50+ years (n = 311)	46.6	47.5	28.6	45.2		
Household head Sex (n = 668)						
Male (n = 435)	65.1	66.2	50.0	58.1	0.150	0.078
Female (n = 233)	34.9	33.8	50.0	41.9		
Household head Education (n = 603)						
None (n = 248)	41.1	41.8	40.0	28.0		
< completed secondary (n = 276)	45.8	45.6	52.0	44.0	0.187	0.722
Secondary & Tertiary (n = 79)	13.1	12.7	8.0	28.0		
Relationship to child (n = 663)						
Parent (n = 283)	42.7	42.9	50.0	32.3		
Grandparent (n = 297)	44.8	44.7	42.9	48.4	0.546	0.623
Other (n = 83)	12.5	12.4	7.1	19.4		
Food Security (n = 629)						
Enough (n = 503)	80.0	79.2	78.6	96.7	0.064	0.940
Not enough (n = 126)	20.0	20.8	21.4	3.3		
SES Tertiles (n = 654)						
Most poor (n = 218)	33.3	33.3	42.9	29.0		
Middle (n = 218)	33.3	34.0	35.7	19.4	0.126	0.392
Least poor (n = 218)	33.3	32.9	21.4	51.6		
**Area of Residence**						
Mainly South African village (n = 621)	92.6	92.1	96.4	96.8	0.461	0.406
Mainly Mozambican village (n = 50)	7.5	7.8	3.6	3.2		

^1^Age at interview date

### Nutritional outcomes

The prevalence of stunting was 18% in this age group (n = 117), but though higher in HIV positive children (29%) than HIV negative children (18%), the difference did not reach statistical significance (p = 0.136). Prevalence of underweight was 10% (n = 67), while that of wasting was 7% (n = 44). There was also no significant difference in the levels of underweight and wasting by HIV status (p = 0.480 and p = 0.389, respectively). Height- and weight-for-age and weight-for-height z-scores were all significantly lower in HIV positive children compared to HIV negative children (p < 0.05) as indicated in the univariate results (Table 3, 4 &5, respectively).

**Table 3:** Regression analysis of determinants of HAZ and Stunting for children aged 12-59 months (n = 671), Agincourt, South Africa (2007)

	HAZ [n = 670]		Stunting [n stunted = 117]	
	**Univariate Coeff** **[95% CI] P**	**Multivariate**^**1 **^**Coeff** **[95% CI] P**	**Univariate OR** **[95% CI] P**	**Multivariate**^**1 **^ **OR [95% CI] P**
**Child Characteristics**				
HIV status (n = 671)				
Negative (n = 612) (ref)	0	0	1	1
Positive (n = 28)	-0.7[-1.1, -0.2] 0.004	**-0.8[-1.2, -0.3] 0.001**	1.9 [0.8, 4.4] 0.142	2.3 [0.9, 5.6] 0.075
Unknown status (n = 31)	0.3 [-0.2, 0.7] 0.249	0.2 [-0.2, 0.6] 0.398	0.3 [0.1, 1.4] 0.128	0.4 [0.1, 1.6] 0.186
Child age (n = 671)	0.2 [0.2, 0.3] <0.001	**0.2 [0.1, 0.3] <0.001**	0.6 [0.5, 0.7]<0.001	**0.6 [0.5, 0.7]<0.001**
Birth weight (n = 522)				
Normal; 2.5 kg (n = 468) (ref)	0	0	1	1
Low; <2.5 kg (n = 54)	-0.6[-0.9, -0.3]<0.001	**-0.6 [-0.9, -0.3]<0.001**	2.1 [1.1, 4.0] 0.023	1.9 [0.9, 3.8] 0.070
**Maternal Characteristics**				
Mother's age (n = 659)				
25-34 (n = 277) (ref)	0	0	1	1
15-24 (n = 207)	-0.4 [-0.6, -0.2] 0.001	**-0.3 [-0.5, -0.1] 0.005**	1.7 [1.1, 2.7] 0.025	1.6 [1.0, 2.6] 0.051
35+ (n = 175)	0.0 [-0.2, 0.2] 0.955	-0.1 [-0.3, 0.2] 0.578	0.8 [0.5, 1.4] 0.501	1.1 [0.6, 1.9] 0.851
Mother co-residence (n = 671)				
Co-residing (n = 650) (ref)	0	0		
Not co-residing (n = 21)	0.6 [0.1, 1.1] 0.019	0.6 [-0.1, 1.2] 0.082	0.5 [0.1, 2.1] 0.340	
**Household Characteristics**				
Relationship to child (n = 663)				
Parent (n = 283) (ref)	0	0		
Grandparent (n = 297)	-0.0 [-0.2, 0.2] 0.892	0.1 [-0.2, 0.3] 0.503	1.0 [0.7, 1.6] 0.871	
Other (n = 83)	-0.3 [-0.6, 0.0] 0.057	-0.2 [-0.5, 0.1] 0.267	1.2 [0.6, 2.2] 0.580	
**Area of Residence **(n = 671)				
Mainly South African Village (n = 621) (ref)	0	0	1	1
Mainly Mozambican Village (n = 50)	-0.5 [-0.8, -0.1] 0.008	-0.3 [-0.7, -0.0] 0.062	2.4 [1.3, 4.6] 0.006	**2.2 [1.1, 4.3] 0.024**

1 Only indipendent variables significantly associated with the outcome variable of interest at the 10% level of significance in the univariate analysis were included in multivariate analysis. Other independent factors examined include child's sex; place of delivery; nationality, highest education level, and marital/union status of the mother; age, sex and highest education level of household head; household food security; and socio-economic status.

### Determinants of child nutritional status

Results of regression analysis are shown on Tables 3, 4 and 5. Only variables that were significantly associated with nutritional status from the univariate analyses at the 10% level of significance are included in the tables.

#### Height- and weight-for-age and weight-for-height z-scores

Key determinants of HAZ were at child level: HIV status, child's age and birth weight; and at maternal level: mother's age (all p < 0.050). HIV positive children had significantly lower HAZ than HIV negative children (p = 0.001); age was positively associated with HAZ (p < 0.001); low birth weight was negatively associated with HAZ (p < 0.001); while children of mother's aged 15-24 years had significantly lower HAZ than of mothers aged 25-34 years (p = 0.005) (Table 3). Key determinants of WAZ were at child level: HIV status and birth weight; at maternal level: mother's age; and at household level: household head's age (all p < 0.050). HIV positive status was negatively associated with WAZ (p = 0.001); low birth weight was negatively associated with WAZ (p < 0.001); children of mother's aged 15-24 years had significantly lower WAZ than of mothers aged 25-34 years (p = 0.011); while children of household heads younger than 35 years had lower z-scores compared to those of household heads in the age-category 35-49 years (p = 0.047) (Table 4). Key determinants of WHZ were only at child level and included child's age and birth weight. Both age and low birth weight were negatively associated with WHZ (p = 0.001 & p < 0.001, respectively). (Table 5)

**Table 4:** Regression analysis of determinants of WAZ and Underweight for children aged 12-59 months (n = 671), Agincourt, South Africa (2007)

	WAZ [n = 671]		Underweight [n underweight= 67]	
	**Univariate Coeff** **[95% CI] P**	**Multivariate**^**1 **^**Coeff** **[95% CI] P**	**Univariate OR** **[95% CI] P**	**Multivariate**^**1 **^**OR** **[95% CI] P**
**Child Characteristics**				
HIV status (n = 671)				
Negative (n = 612) (ref)	0	0	1	1
Positive (n = 28)	-0.7 [-1.2, -0.3] 0.001	**-0.7 [-1.2, -0.3] 0.001**	1.5 [0.5, 4.4] 0.482	1.5 [0.5, 4.4] 0.512
Unknown status (n = 31)	0.3 [-0.1, 0.7] 0.137	0.2 [-0.2, 0.6] 0.137	0.3 [0.0, 2.2] 0.235	0.3 [0.0, 2.2] 0.228
Birth weight (n = 522)				
Normal; 2.5 kg (n = 468) (ref)	0	0	1	1
Low; <2.5 kg (n = 54)	-0.8 [-0.1, -0.5] <0.001	**-0.7 [-1.1, -0.4] <0.001**	3.1 [1.5, 6.5] 0.002	**3.1 [1.5, 6.4] 0.002**
**Maternal Characteristics**				
Mother's age (n = 659)				
25-34 (n = 277) (ref)	0	0		
15-24 (n = 207)	-0.2 [-0.4, -0.0] 0.044	**-0.3 [-0.5, -0.1] 0.011**	1.0 [0.6, 1.8] 0.955	
35+ (n = 175)	0.0 [-0.2, 0.2] 0.979	-0.1 [-0.3, 0.2] 0.633	0.9 [0.5, 1.6] 0.647	
Mother's Education (n = 625)				
None (n = 83) (ref)	0	0		
Some education <completed secondary (n = 381)	0.2 [-0.1, 0.4] 0.267	0.2 [-0.1, 0.5] 0.269	1.1 [0.6, 2.0] 0.756	
Secondary & Tertiary (n = 191)	0.4 [0.1, 0.8] 0.007	0.3 [-0.0, 0.6] 0.085	0.7 [0.3, 1.5] 0.362	
Delivery place (n = 671)				
Health facility (n = 568) (ref)	0	0		
Home (n = 103)	-0.3 [-0.5, -0.1] 0.019	-0.2 [-0.5, -0.1] 0.284	1.2 [0.6, 2.4] 0.541	
**Household Characteristics**				
Household head Age (n = 668)				
35-49 (n = 283) (ref)	0	0		
15-34 (n = 74)	-0.4 [-0.7, -0.1] 0.022	**-0.3 [-0.6, -0.0] 0.047**	1.8 [0.8, 3.9] 0.129	
50+ years (n = 311)	-0.2 [-0.3, 0.0] 0.124	-0.1 [-0.3, 0.7] 0.228	1.1 [0.7, 2.0] 0.637	
SES Tertiles (n = 654)				
Most poor (n = 218) (ref)	0	0		
Middle (n = 218)	0.2 [-0.1, 0.4] 0.172	0.0 [-0.2, 0.3] 0.690	0.6 [0.3, 1.2] 0.143	
Least poor (n = 218)	0.3 [0.1, 0.5] 0.012	0.1 [-0.1, 0.3] 0.500	1.0 [0.2, 4.8] 0.971	
**Area of Residence **(n = 671)				
Mainly South African Village (n = 621) (ref)	0	0	1	1
Mainly Mozambican Village (n = 50)	-0.5 [-0.8, -0.1] 0.006	-0.3 [-0.7, 0.0] 0.079	2.1 [1.0, 4.6] 0.054	2.0 [0. 9, 4.4] 0.094

1 Only indipendent variables significantly associated with the outcome variable of interest at the 10% level of significance in the univariate analysis were included in multivariate analysis. Other independent factors examined include child's sex and age; child's relationship to household head; nationality, and marital/union status of the mother; mother's co-residence with child; sex and highest education level of household head; and household food security

**Table 5:** Regression analysis of determinants of WHZ and wasting for children aged 12-59 months (n = 671), Agincourt, South Africa (2007)

	WHZ [n = 670]		Wasting [n wasted = 44]	
	**Univariate Coeff** **[95% CI] P**	**Multivariate**^**1 **^**Coeff** **[95% CI] P**	**Univariate ** **OR [95% CI] P**	**Multivariate**^**1 **^ **OR [95% CI] P**
**Child Characteristics**				
HIV status (n = 671)				
Negative (n = 612) (ref)	0	0	1	1
Positive (n = 28)	-0.5 [-1.0, -0.0] 0.047	-0.5 [-1.0, 0.1] 0.094	1.7 [0.5, 5.9] 0.395	1.9 [0.5, 6.5] 0.333
Unknown status (n = 31)	0.2 [-0.3, -0.1] 0.322	0.2 [-0.3, 0.7] 0.338	0.48 [0.1, 3.6] 0.471	0.5 [0.07, 4.1] 0.560
Child age (n = 671)	-0.2 [-0.3, -0.1] 0.001	**-0.2 [-0.3, -0.1] 0.001**		
Birth weight (n = 522)				
Normal; 2.5 kg (n = 468) (ref)	0	0		
Low; <2.5 kg (n = 54)	-0.7 [-1.1, -0.3]<0.001	**-0.7 [-1.0, -0.3]<0.001**	1.8 [0.7, 4.9] 0.248	
**Maternal Characteristics**				
Mother's Education (n = 625)				
None (n = 83) (ref)	0	0		
Some education <completed secondary (n = 381)	0.2 [-0.1, 0.5] 0.175	0.2 [-0.2, 0.5] 0.302	0.8 [0.4, 1.6] 0.459	
Secondary & Tertiary (n = 191)	0.3 [-0.0, 0.7] 0.081	0.2 [-0.2, 0.6] 0.300	0.8 [0.4, 2.0] 0.669	
Delivery place (n = 671)				
Health facility (n = 568) (ref)	0	0	1	1
Home (n = 103)	-0.4 [-0.7, -0.1] 0.010	-0.2 [-0.6, -0.1] 0.149	2.2 [1.1, 4.4] 0.027	2.0 [1.0, 4.1] 0.059
**Household Characteristics**				
Household head Age (n = 668)				
35-49 (n = 283) (ref)	0	0		
15-34 (n = 74)	-0.4 [0.7, -0.0] 0.034	-0.3 [-0.7, -0.0] 0.066	1.7 [0.6, 4.6] 0.301	
50+ years (n = 311)	-0.2 [-0.4, 0.0] 0.086	-0.2 [-0.4, -0.0] 0.088	1.6 [0.8, 3.2] 0.175	
SES Tertiles (n = 654)				
Most poor (n = 218) (ref)	0	0		
Middle (n = 218)	0.1 [-0.1, 0.4] 0.363	0.1 [-0.2, 0.3] 0.541	0.9 [0.4, 1.8] 0.714	
Least poor (n = 218)	0.3 [0.0, 0.5] 0.038	0.2 [-0.1, 0.4] 0.241	0.6 [0.3, 1.4] 0.250	
**Area of Residence **(n = 671)				
Mainly South African Village (n = 621) (ref)			1	1
Mainly Mozambican Village (n = 50)	-0.3 [-0.7, 0.1] 0.113		2.6 [1.1, 6.1] 0.033	2. 3 [0.9, 5.5] 0.070

1 Only indipendent variables significantly associated with the outcome variable of interest at the 10% level of significance in the univariate analysis were included in multivariate analysis. Other independent factors examined include child's sex; child's relationship to household head; age, nationality and marital/union status of the mother; mother's co-residence with child; sex and highest education level of household head and household food security.

#### Stunting, underweight and wasting

Key determinants of stunting included child's age, and area of residence. Age was negatively associated with stunting (p < 0.001); while children living in villages mainly inhabited by people of Mozambican origin had more than two-fold higher odds of being stunted (p = 0.024). Association of stunting with mother's age was borderline (p = 0.051), with children born to mothers younger than 25 years having a 1.6 higher odds of being stunted than children born to older mothers aged 35-49 years (Table 3). Only low birth weight was a significant predictor of underweight: children with low birth weight had three-fold higher odds of being underweight than other children (p = 0.002) (Table 4). No factor was significantly associated with wasting (Table 5).

### Summary of determinants

Key predictors of nutritional status in children aged 12-59 months in Agincourt include child's HIV status, child's age, birth weight, maternal age, age of household head, and area of residence. HIV status was strongly associated with z-scores but its association with stunting, underweight and wasting was not significant at 5% level of significance.

## Discussion

This study has described the prevalence of HIV, nutritional outcomes by HIV status, and determinants of nutritional status among children 12-59 months living in rural South Africa. Nutritional status was determined using height- and weight-for-age and weight-for-height z-scores. HIV positive children had poorer nutritional outcomes compared to their HIV negative counterparts. In addition to HIV, important covariates of children's nutritional status at the child, household and community level were detected. At child level, these included child's age and birth weight; at maternal level, maternal age; at household level, age of household head; and at community level, area of residence.

The HIV prevalence in this study is seemingly low and is comparable to 2005 national estimates among children aged 2-4 years (5%) in South Africa [30], despite high HIV prevalence among women of child-bearing age [13]. Prevention of mother to child transmission (PMTCT) programs have been scaled up in South Africa in the last few years and some progress has been made for example with regards to antenatal HIV testing and uptake of nevirapine for infected mothers [31]. This prevalence of HIV in children is however important both for individual wellbeing of the children and for public health purposes. HIV infection influences nutritional outcomes of infected children at an individual level [6], while malnutrition in HIV infected children leads to higher mortality [7]. HIV in children has been associated with remarkable demographic impacts in South Africa. Dorrington et al. (2006) estimated that 44% of mortality among children aged 0-14 years was due to HIV despite only 2% of this age group being infected [32]. The higher prevalence of HIV observed in girls compared to boys cannot be fully explained in this study, but may result from longer survival of HIV positive girls. Although numbers are small, during a one year follow-up of the HIV positive children, two boys died and no girls [33].

This study documents a high prevalence of undernutrition, particularly stunting, which is more prevalent in HIV positive children. The higher vulnerability of HIV positive children to poorer nutritional outcomes in this study may be influenced by their HIV status, since after controlling for other covariates of nutritional status in children, HIV status remained a significant determinant of many of the outcomes. Growth failure has been associated with HIV elsewhere in sub-Saharan Africa [7,34] and in South Africa in particular [35]. In settings with unhygienic environments and poor water, infections in young children - particularly diarrhoea - are important causes of undernutrition [36]. Recurrent illnesses such as diarrhoea, exacerbated in HIV positive children due to lowered immunity, may play a major role in poor nutritional outcomes. This study also documented other vulnerabilities that may jeopardize the nutritional status of HIV positive children relative to their HIV negative counterparts. These include lower birth weight, lower maternal education and less likelihood of maternal co-residence with child mainly due to death.

Consistent with other studies in developing countries [10], child level factors including birth weight and child's age emerged as key. Though further investigations are needed to establish the causes of low birth weight in this setting, one factor may be maternal health and the impact of HIV exposure, as a third of pregnant women visiting public health facilities are HIV infected [13]. Maternal HIV infection which leads to higher rates of maternal opportunistic infections has particularly been associated with foetal growth retardation leading to smaller size and low birth weight [37]. Birth weight was lower in HIV positive children compared to their negative counterparts as also found in other studies in sub-Saharan Africa [38]. Child age was differentially related to nutritional status. With regards to height-for-age z-scores and stunting, indicators for chronic malnutrition, older children had better outcomes, while with regards to weight-for-height z-scores, older children had poorer outcomes. The height-for-age z-scores and higher stunting in younger children may be associated with low birth weight, followed by catch up growth in the older years. The poorer outcomes related to weight-for-height z-scores in older children may be associated with feeding practices, which may be associated with compromised hygienic practices, hence infections [39-41].

The maternal level factor found to be significantly associated with child's nutritional status was maternal age. Maternal age has been documented in other studies in the developing world as an important risk factor for child malnutrition [11]. The increased risk of malnutrition in children of younger mothers may relate to inexperience and inadequate child care, or to biological characteristics such as small maternal size with potential for low birth weight and later poor nutritional outcomes.

At the household level, only age of the household head emerged as a predictor of nutritional outcomes associated with both weight-for-age and weight-for-height z-scores. The pathway through which age of the household head may influence nutritional outcomes of the child could be through income and food security of the household. Contrary to expectation, food insecurity did not emerge as an important factor with regards to nutritional status of children in this age category. The lack of association found between food (in)security and nutritional outcomes may relate to food (in)security being a household-level measurement and thus less sensitive than individual-level measurements of child's food intake. Additionally, poor nutritional outcomes may not only be associated with the quantity of food but also the quality, which this study did not assess. Food variety and dietary diversity are limited in those living in poor socio-economic circumstances in South Africa and are associated with nutritional status of South African children [42]. Additionally, socio-economic status, a factor associated with nutritional outcomes in other studies in the developing world [43], was not a significant determinant of nutritional outcomes after controlling for other covariates. This suggests that other factors may be more important in determining the nutritional status of young children in this community. Socio-economic status was estimated using a household asset index as a proxy measure of relative household wealth rather than actual income or expenditure levels, and may not necessarily relate to households' current socio-economic status. However, this method has been shown as valid elsewhere [21].

At the community level, area of residence emerged as a predictor of nutritional outcomes. Area of residence serves as a proxy for various factors at community level, including environmental factors, availability of health care and support services, and shared cultures. Children living in villages mainly inhabited by people of Mozambican origin had poorer nutritional outcomes. These villages served as refugee settlements during and after the civil war in Mozambique from the early to mid-1980s. Consequently, well over 90% of people living in these villages are of Mozambican origin, whereas they constitute about 30% of inhabitants over the study site as a whole. Though the situation is changing, people in these villages have lived with limited legal recognition without fully being integrated into South African society. The villages have poor dwellings and infrastructure and are worse off than mainly South African villages with respect to basic services including water, sanitation, electricity and health facilities [44,45]. Other researchers in the study area have also found poorer health outcomes in children living in former Mozambican households [46]. While this study showed that being born to a mother of Mozambican origin does not disadvantage the child with regards to nutritional outcomes, previous work documented significantly higher mortality in children born to Mozambican mothers compared to those born to South African mothers [47]. Community interventions to improve living conditions could contribute to better health and nutritional outcomes in children.

A few limitations of this study are important to note. About a quarter of sampled children did not participate in the study. Though there was no significant variation for most demographic variables examined, non-participants were significantly younger, had younger mothers and were more likely to have a grand-parent as the head of household. Since the study shows that younger children and children of younger mothers were more likely to be stunted and had lower height- and weight-for-age z-scores, non-participation may have underestimated the reported prevalence of stunting and overestimated the reported height- and weight-for-age z-scores. The sample was drawn from the existing Agincourt HDSS, hence the time lag between data collection for the Agincourt HDSS sampling frame and data collection for the growth survey meant that infants under one year of age were not included. This limitation may have lowered the proportion of children with HIV infection reported in our study, and masked the extent of poor nutritional outcomes in HIV positive children as the most vulnerable children may have died by their first birthday [48]. Further, the bivariate results by HIV status should be interpreted with caution as the small proportion of HIV positive children in our study may bias the associations, in that we may have failed to detect significant associations based on proportions, which may have existed. A further limitation relates to the HIV tests employed: according to WHO recommendations, antibody tests are appropriate for HIV screening in children aged 18 months and above. As maternal antibodies may still be present in younger children, assays to detect the virus rather than antibodies are recommended for children younger than 18 months [29]. However, most uninfected HIV-exposed children have lost maternal antibodies by 12 months of age; hence an HIV antibody-positive test at this age may thus be considered indicative of HIV infection [29,49]. Since only three children under 18 months of age tested HIV positive in our study, the likelihood of maternal antibodies affecting the results is low. We did not use an elaborate food security status assessment as the tool did not include diverse dimensions of food security. This may have implications on our results, such as the observed lack of association of food security status with nutritional outcomes. Despite these limitations, a few strengths of the study need mention. These relate to the purely random sampling strategy employed and the high response rate for the HIV test (95%). The small proportion of non-response for the test was mainly due to labour migration (of decision makers), which, mainly circular rural-urban migration, is widespread in the area and characterise such rural communities of South (and southern) Africa [17,18].

The findings are important and reinforce the need to focus on the nutritional status of all young children in rural communities, including those infected or exposed to HIV. Effective maternal interventions on nutrition and care of young children are needed. Further, the higher risk of poor nutritional outcomes in children with very young mothers indicates need for community- and school-based education to reduce early pregnancies, and post-school training and job opportunities particularly for girls. We further recommend interventions targeting development of more disadvantaged areas, including improved provision of basic services such as water and sanitation, electricity and other basic infrastructure. Additionally, improved legal and social integration of people of Mozambican origin for example through accelerated provision of identity documents for those who lack these may improve the nutritional status of their children through prompt access to government social grants such as child support grants.

Interventions to improve nutritional outcomes of children infected or exposed to HIV may include improving access to and utilization of PMTCT services and targeted paediatric HIV screening and support to infected children. Despite scale-up of PMTCT programs in South Africa in the last few years [31], access to and utilization of these services remain a challenge particularly in rural areas [50,51] and are in urgent need of improvement. Where PMTCT has failed, early detection of HIV positive children is critical for appropriate medical management, reduction of morbidity and mortality and improvement of quality of life [29]. The high level of paediatric HIV test uptake in the study indicates that rapid tests for HIV screening in children are acceptable in this rural community. Further, in a one year qualitative follow-up study [33], most caregivers of HIV positive children reported a positive response to knowing the child's status. This knowledge was said to heighten caregiver's own competency in caregiving and health care seeking. Based on these findings, the negative impact of HIV on the growth of infected children [7,34], and the estimated high impact of HIV infection on child mortality [32], we recommend paediatric HIV screening at community level for those exposed to HIV or suspected to be at risk for HIV. This may be done using rapid tests with appropriate follow-up measures in the presence of HIV antibodies including definitive testing and support to the mother/caregiver and her family including counselling [29]. Interventions targeted specifically at HIV positive children could include food supplementation [34] and antiretroviral treatment [52].

## Conclusion

This study documents poor nutritional status among children aged 12-59 months and heightened nutritional vulnerability in HIV positive children compared to their HIV negative counterparts. Early paediatric HIV testing of exposed or at risk children, followed by appropriate medical care including antiretroviral treatment and nutritional supplementation to infected children may improve their nutritional status. In addition to HIV status, other child, maternal and community-level characteristics emerge as strong determinants of nutritional outcomes in these children. Interventions that effectively counter malnutrition are paramount if we are to address the sequelae of poor child nutrition: poor cognitive development, reduced human capital, premature mortality and other health consequences [2-4].

## Abbreviations

HDSS: Health and socio-demographic surveillance system; AIDS: Acquired immune deficiency syndrome; ANOVA: Analysis of variance; ART: Anti-retroviral therapy/treatment; CI: Confidence interval; HAZ: Height-for-age z-scores; HIV: Human immunodeficiency virus; Kg: Kilogram; MDGs: Millennium development goals; P: Probability; PMTCT: Prevention of mother to child transmission of HIV; OR: Odds ratio; SD: Standard deviation; SES: Socio-economic status; USD: United States dollar; WAZ: Weight-for-age z-scores; WHO: World Health Organization; WHZ: Weight-for-height z-scores

## Competing interests

The authors declare that they have no competing interests.

## Authors' contributions

**EWK-M**: Design of the study, project implementation and management, data management and analysis, writing of the manuscript. Read and approved the final manuscript. **SAN**: Design of the study, overall project management, review of the manuscript. Read and approved final the manuscript. **JMP**: Design of the study, review of the manuscript. Read and approved the final manuscript. **SMT**: Design of the study, review of the manuscript. Read and approved the final manuscript. **KK-G**: Analytic guidance, review of the manuscript. Read and approved the final manuscript. **DD**: Design of the study, review of the manuscript. Read and approved the final manuscript. **FXG**: Project implementation and management, review of the manuscript. Read and approved the final manuscript. **KK**: Design of the study, overall project co-ordination, review of the manuscript. Read and approved the final manuscript.

## Pre-publication history

The pre-publication history for this paper can be accessed here:

http://www.biomedcentral.com/1471-2431/11/23/prepub
